# Swordfish bill injury involving abdomen and vertebral column: case report and review

**DOI:** 10.1186/1471-2482-10-30

**Published:** 2010-10-22

**Authors:** Despoina Georgiadou, George N Zografos, Dennis Vaidakis, Spiridon Avlonitis, Angeliki Katopodi, Emmanouil N Tzirakis, Panagiotis Sioutos, Charalambos Drossos, Penelope Lampropoulou, George Papastratis

**Affiliations:** 1 3rd Surgical Clinic of George Gennimatas General Hospital, Mesogeion Ave 154, 156 69 Athens, Greece; 2 Department of Neurosurgery of George Gennimatas General Hospital, Mesogeion Ave 154, 156 69 Athens, Greece; 3 Department of Radiology of George Gennimatas General Hospital, Mesogeion Ave 154, 156 69 Athens, Greece

## Abstract

**Background:**

Penetrating injuries of the abdomen and spinal canal that involve organic material of animal origin are extremely rare and derive from domestic and wild animal attacks or fish attacks.

**Case presentation:**

In this case report we present the unique, as far as the literature is concerned, unprovoked woman's injury to the abdomen by a swordfish. There are only four cases of swordfish attacks on humans in the literature - one resulted to thoracic trauma, two to head trauma and one to knee trauma, one of which was fatal - none of which were unprovoked. Three victims were professional or amateur fishermen whereas in the last reported case the victim was a bather as in our case. Our case is the only case where organic debris of animal's origin remained in the spinal canal after penetrating trauma.

**Conclusions:**

Although much has been written about the management of penetrating abdominal and spinal cord trauma, controversy remains about the optimal management. Moreover, there is little experience in the management of patients with such spinal injuries, due to the fact that such cases are extremely rare. In this report we focus on the patient's treatment with regard to abdominal and spinal trauma and present a review of the literature.

## Background

Penetrating injuries, although less common than blunt trauma due to motor vehicle accidents, are important causes of injury to the abdomen and vertebral column.

Civilian penetrating injuries are usually related to road traffic, labor and domestic accidents, violent actions, iatrogenic impaction of dental and surgical materials and instruments, and certain types of sports. The majority of civilian deep penetrating foreign body injuries described in the literature involve wood and metal objects.

There are only four cases of swordfish attacks on humans in the literature - one resulted to thoracic trauma [1], two to head trauma [2,3] and one to knee trauma [4], one of which was fatal - none of these were unprovoked. Three victims were professional or amateur fishermen and one victim was a bather. We present here a unique, according to the literature, case of a woman who was injured in the abdomen by a swordfish. It is the only case of unprovoked swordfish attack on humans.

## Case Presentation

A 31-year-old woman was admitted to our hospital emergencies referred from the Medical Center of Santorini Island with a preliminary diagnosis of acute abdomen, after a penetrating trauma in the upper right abdomen by a swordfish. Whilst she was swimming close to the seacoast, only three meters away from the beach yet in waist-deep water, she felt a stab to her right hypochondrial area. After the first shock, she realized it was a fish that had attacked her and she pulled it out of her body. Neither she nor any other bather swimming nearby saw any fish approaching her. However, after her injury, some of the bathers dived and saw the fish running away towards deeper water. The patient was withdrawn from the sea and, having received first aid by the local lifeguard, she was immediately transferred to the island's Medical Center by her companions. Witnesses' testimony, which was enforced by a part of the fish bill, measured to be 20 cm, found at the bottom of the sea in the area of the incident by the Coast Guard of Santorini Island led ichthyologists to identify the fish as a billfish, Xiphias gladius (swordfish).

**Figure 1:**
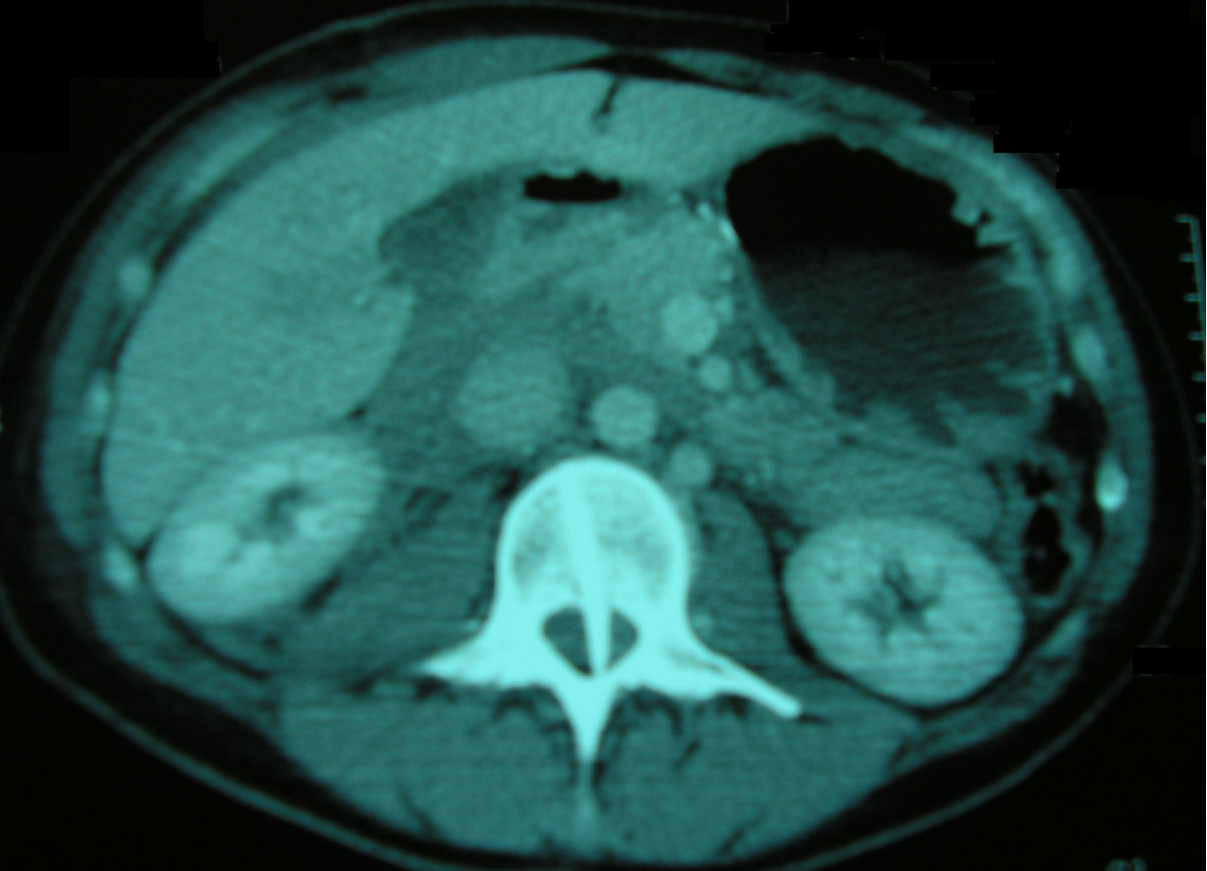
**Abdominal computed tomography scan on admission, demonstrating a penetrating foreign body**.

**Figure 2:**
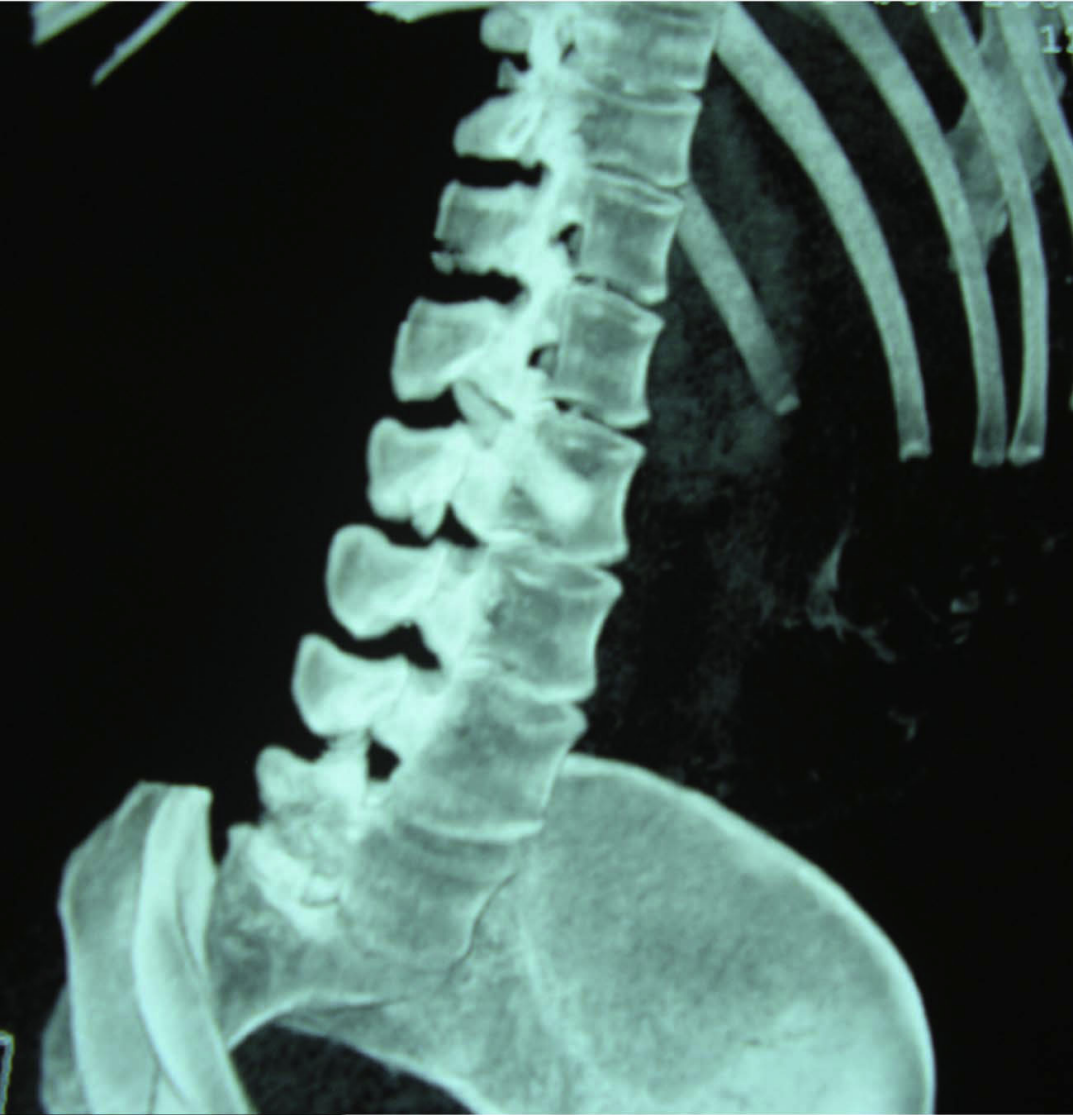
**CT reconstruction image demonstrating the tip of the swordfish bill lodged within the spinal canal**.

**Figure 3:**
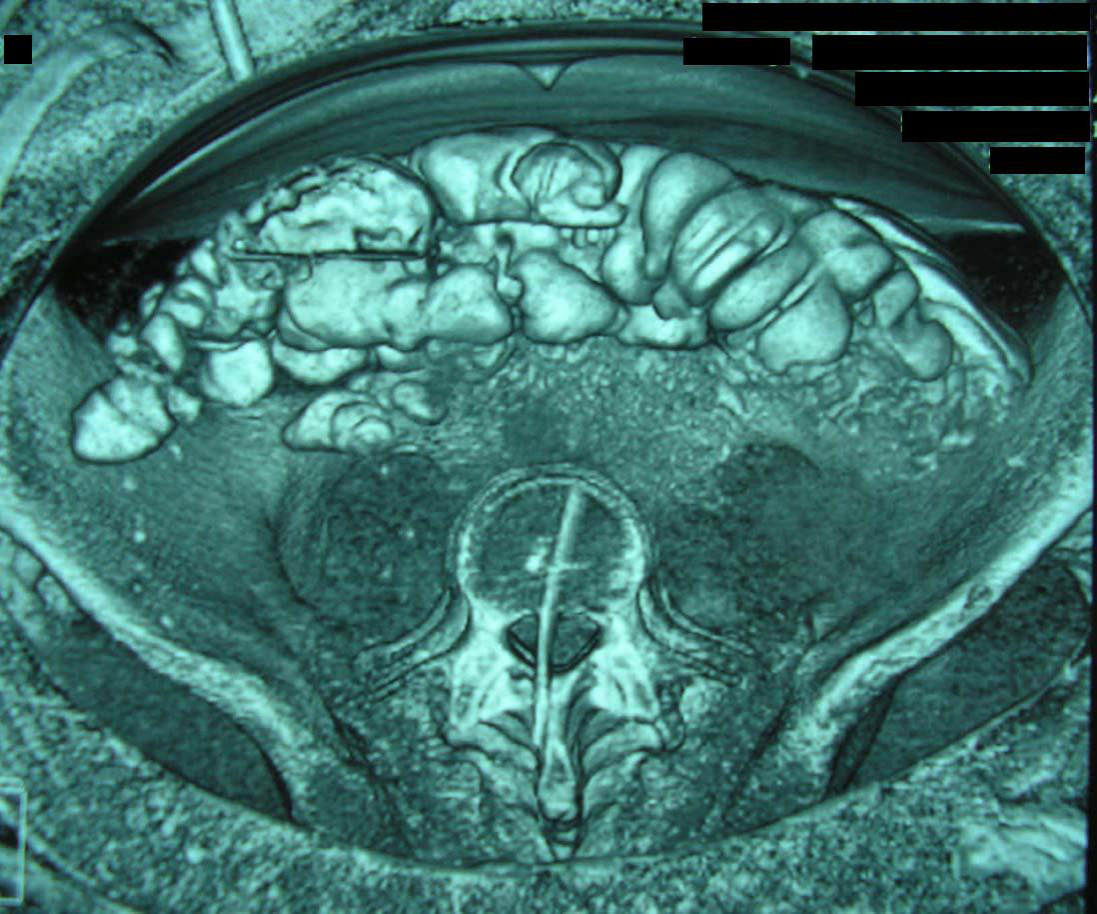
**CT reconstruction image demonstrating the tip of the swordfish bill lodged within the spinal canal**.

**Figure 4:**
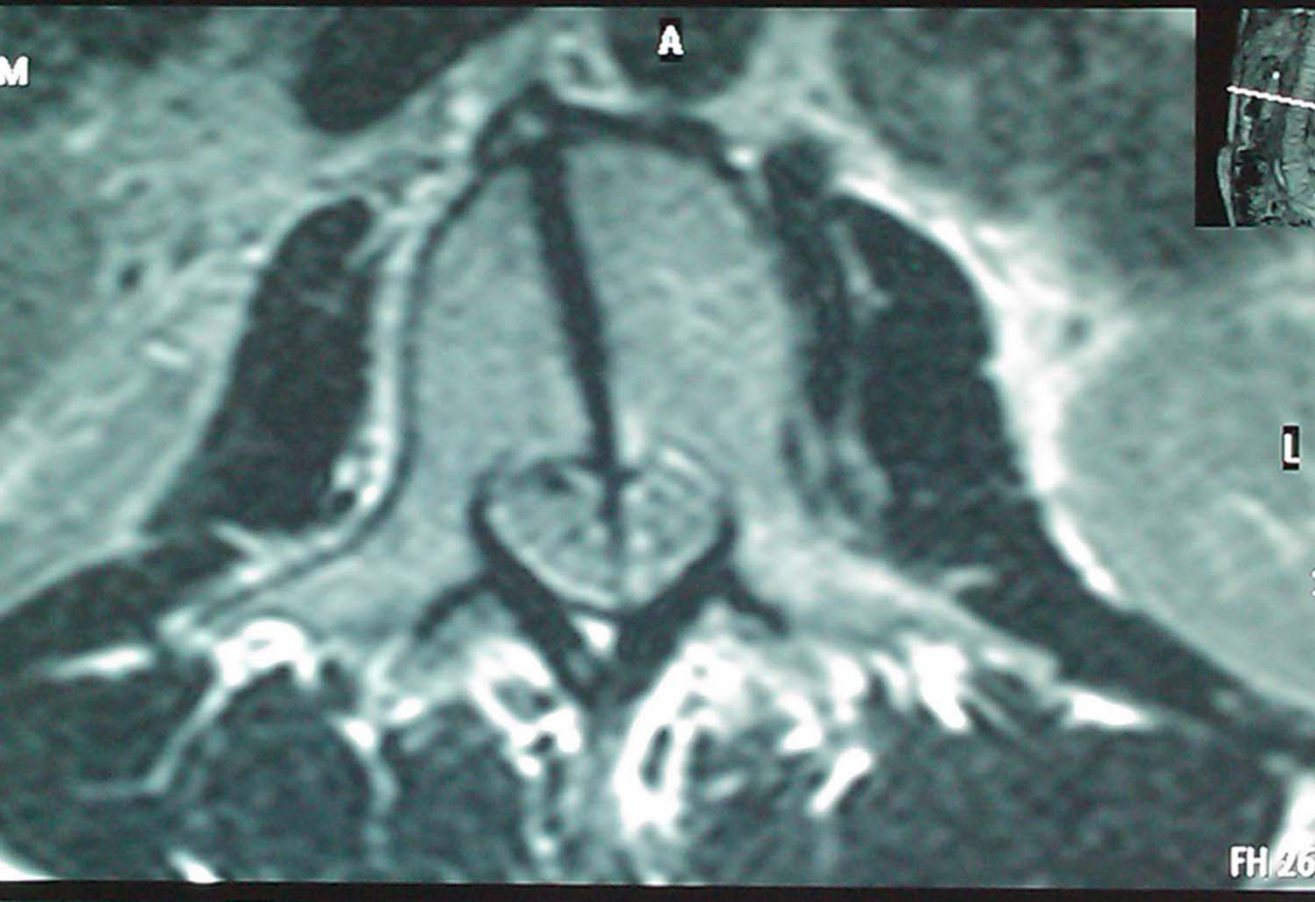
**MRI scan of the lumbar region that indicates a foreign body penetrating the body of the L2 and the spinal canal**.

Her vital signs on presentation to our hospital were: blood pressure 113/71 mm Hg, heart rate 81 beats/minute, arterial blood oxygen saturation 100% and temperature 38.6°C. Physical examination was remarkable only in the abdomen which was scaphoid, tender to palpation with diminished bowel sounds and a 4 cm trauma at the mid-axillary line below the right costal margin. The patient received a broad-spectrum antibiotic and a prophylactic tetanus toxoid injection. Laboratory tests showed a haematocrit of 28.5%, haemoglobin levels of 9.7 g/dL, white blood cell count of 25 × 10^9^/L (with 94.10% polymorphonuclear cell type), and platelet count of 152 × 10^9^/L. The coagulation profile was within normal limits. Thoracic and abdominal X-rays performed at the island's Medical Center did not reveal any pathology whilst F.A.S.T (Focused Assessment Sonography in Trauma) performed there marked out free intraperitoneal fluid at the right circumrenal and Duglas areas. An abdominal Computed Tomography (CT) scan in our hospital revealed: laceration of right liver lobe, distension of inferior vena cava, distension of right renal vein and a hyperdense bone-type foreign body (swordfish bill tip) retroperitoneally. The latter had entered the spinal canal through the body of the second lumbar vertebra (L2) after crossing the abdominal cavity (Figure 1). CT reconstruction images demonstrating the tip of the swordfish bill lodged within the spinal canal were produced (Figure 2, Figure 3). The magnetic resonance imaging (MRI) scan of the lumbar region indicated a longitudinal foreign body, which had reached the interspinous space of the second and third lumbar spinous processes, through the body of the L2 and the spinal canal (Figure 4). Myelography showed that the foreign body penetrated tabular roots and spinal canal at the area mentioned above and further neurological examination was recommended. The neurological (sensory and mobility) examination of both legs, as well as the sensory examination of the perineum did not reveal any neurological deficit.

After all diagnostic tests had been completed and interpreted, the patient was transfused with one unit of blood and assigned to surgery for intra-abdominal bleeding. Penetration of the abdomen was present at the mid-axillary line, below the right costal margin. The abdomen was explored through bilateral subcostal incision. Approximately 1 litre of blood was aspirated from the abdominal cavity and a large right retroperitoneal hematoma was identified. The right liver lobe was mobilized and the portal triad was controlled with a sling. Liver had been penetrated from segment IVa posteriorly and the inferior vena cava (IVC) had been lacerated medially. The duodenum was mobilized and the retroperitoneum was opened. Blood and clots were evacuated from the retroperitoneal space and the IVC was repaired with proline 4-0. Cholecystectomy was performed at this stage because of laceration of the cystic duct. The gastrocolic ligament was subsequently divided and the pancreas and aorta were explored. An unsuccessful effort was done in order to find the foreign body (sword fish bill tip) at the level of L2 vertebra, which was revealed at the CT and MRI scan. A penrose drainage was placed below the liver and the wound was closed.

**Figure 5:**
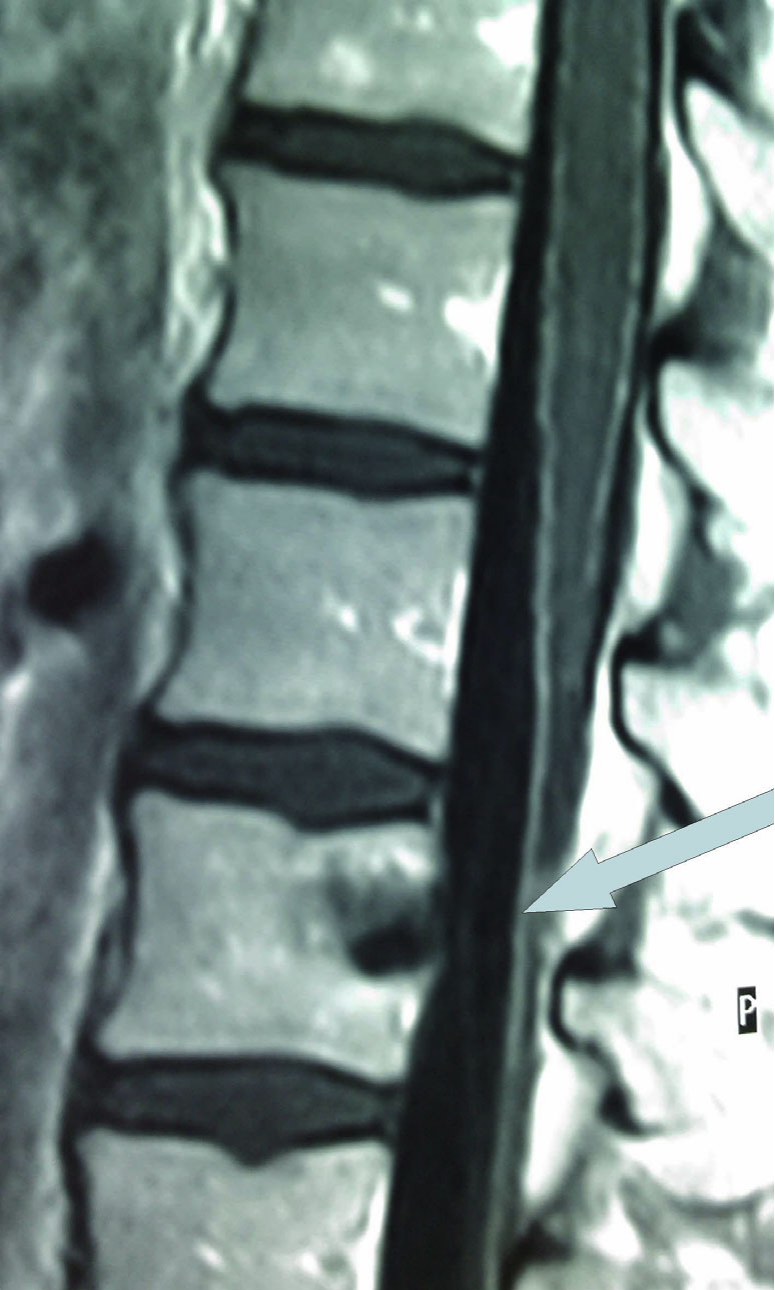
**MRI scan of lumbar region which indicates L2 inflammation and arachnoiditis of some tabular roots**.

**Figure 6:**
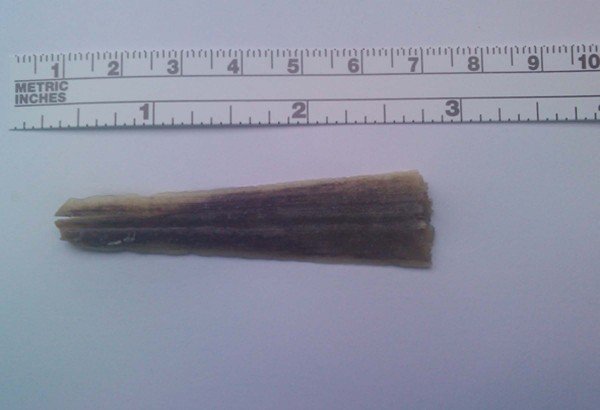
**The fragment (swordfish bill tip) removed**.

After the completion of the surgery, the patient was transferred to the Intensive Care Unit for monitoring and planning of the foreign body removal in future. She was hospitalized there for three days. The patient's clinical state and laboratory parameters were evaluated from orthopedics of our hospital who recommended the patient's mobilization. After that she was transferred to the Third Surgical department for further medical attendance and laboratory tests. On the third postoperative day, she exhibited fever up to 38.5°C which was resistant to antibiotic therapy. Blood cultures were taken and lumbar puncture was performed; the collected sample of cerebrospinal fluid was sent for biochemical and microbiological analysis. Both examinations were negative for bacterium growth. The new thoracic and abdominal computed tomography (CT) scan did not show significant changes compared with CT on her admission to the emergency with the exception of subcapsular fluid collection of increased density at left liver lobe, which was found increased in new CT examination 4 days later. CT- guided drainage of that fluid, which was a biloma, was performed and drainage tube was left at the collection's area. The Magnetic resonance cholangiopancreatography (MRCP), that was performed three days later, did not reveal any pathology of bile ducts or bile leak. The new MRI scan of lumbar region indicated L2 inflammation and arachnoiditis of some tabular roots (Arrow; Figure 5). The patient then was transferred to the Neurosurgical department of our hospital and underwent an operation in order to remove the swordfish bill tip by approaching it from the spinal canal (posteriore approach). Laminectomy of the second and third lumbar vertebra was accomplished and the foreign body was removed intact easily (Figure 6). There was L2 vertebral decomposition as a result of local osteomyelitis. Osteomyelitis of these vertebras was the result of bone infection by bacteria of the epithelium of swordfish bill which invaded the bone directly after the swordfish attack. Consequently, when the cause of osteomyelitis - tip of swordfish bill - removed surgically, patient's fever declined since the first postoperative day and the overall condition improved significantly. Patient was mobilized on the third postoperative day. However, she was hospitalized further in order to receive intravenous antibiotic therapy. She left the hospital on the thirty fifth postoperative day, fourteen days after the abdominal drainage was taken out. Antibiotics were continued for further four more weeks.

Written informed consent was obtained from the patient for the publication of the case report.

## Discussion

According to the literature, the majority of deep penetrating abdominal and spine injuries are provoked by objects made of metal and wood. The most widely reported cause of penetrating injury to spine is gunshot injury [5], with few reports of such injuries involving knifelike objects [6] and glass [7]; only two cases of penetrating injury by wooden fragments into the lumbar spinal canal with [8] and without cauda equina syndrome have been described [6]. Abdominal penetrating trauma is mainly caused by low or high velocity projectiles [9]. Injuries of the abdomen and the spinal canal that involve organic material of animal origin are extremely rare and derive from domestic or wild animal attacks (wild boar's gore [10], cow's gore [11], bull's horn [12], zebu's gore [13]), as well as fish attacks (stingray [14], garfish [15], sturgeon [16] and swordfish [1-4]) according to the literature. To our knowledge, there are only three reports of a swordfish attack on human in the literature that resulted in presence of biological material of such origin in the human body - one in craniofacial structures [3], one in the thorax [1], and one in knee [4]. However, we have to point out that our case is unique due to the reasons below:

- It is the first case of unprovoked attack of swordfish on humans. The three first victims were professional or amateur fishermen whereas in the last reported case the victim was a bather as in our case. However, the bather provoked the swordfish to attack him by mistakenly identifying it to be a shark threatening his child. On the contrary, our patient did not provoke the swordfish in any way since she was swimming carefree and she did not even realise the fish presence until its attack.

- It is the first case of deep abdominal penetrating trauma caused by swordfish attack

- It is the first case of penetrating injury to the spinal canal by bony fragments of this nature- in the case with the penetrating thoracic wound the tip of the sword was adhered to the body of third thoracic vertebra (T3), but there was no bony debris in the spinal canal.

The salient feature of the swordfish is the prolongation of its upper jaw into a long, flat, sword-like bill occupying nearly one-third the total length of the fish [17]. Swordfish, except young fry, are both toothless and scaleless and usually grow to a great size; its common length is 300 cm with maximum cited length 455 cm [18,19]. Maximum published weight is 650 kg [20]. Swordfish is oceanic, not dependent in any way either on the coast, except as this offers a supply of food, or on the bottom; and it is a warm-water fish [18]. They are always seen swimming alone or at most two fishes together. They eat once a day, mainly at night when they rise to surface and near-surface waters surging smaller fishes. They have often been described as rising through schools of fishes, thrashing their swords to harm or kill, and then turning to consume the dead or mangled fish [18,21]. Till now, there were no reports of unprovoked swordfish attack to human. However, there are many reports of swordfish attacks to fishermen and their vessels after the former had been harpooned [22].

Trauma is the leading cause of death and disability during the most productive years of life (25-40) [23]. The liver is the first most commonly injured intra-abdominal organ and is found to be damaged in 30% of patients undergoing laparotomy for penetrating abdominal trauma. The outcome after liver trauma has been shown to be dependent on the severity of the liver injury, the mechanism, and presence of any other associated injuries with a commonly quoted overall mortality of around 12% [24].

The primary goal in the treatment of severe abdominal liver injuries is to preserve life. Management, which has evolved significantly over the last three decades and is based on well defined protocols, is divided into four sequential phases: resuscitation, evaluation, initial management, and definitive treatment. In this case initial resuscitation performed in Medical Center of Santorini Island. The patient received intravenous fluids and broad-spectrum antibiotic therapy which was continued in our hospital.

The key underlying determinant of subsequent management is hemodynamic stability. In cases of abdominal injuries, where hemodynamic stability is maintained with or without volume resuscitation prompt radiological imaging should be performed. Our patient, as mentioned above, underwent X-rays and FAST at island's Medical Center and CT in our hospital.

After initial resuscitation and investigation the question that arises is whether the patient should be managed operatively or non-operatively. Those patients who remain in shock after vigorous intravenous fluid resuscitation usually have continued bleeding and require urgent laparotomy. Surgery should not be delayed to obtain the results of special examinations. Along with haemodynamic instability other major indications for urgent laparotomy are stab or gunshot wounds that have penetrated the abdomen, signs of peritonitis, unexplained shock, evisceration, uncontrolled haemorrhage, clinical deterioration during observation [25]. In our case patient underwent surgical management due to her clinical deterioration during observation and to her presentation with signs of peritonitis three hours after her injury, despite the fact that was hemodynamically stable.

Follow-up scans within 2 to 5 days can determine changes in the injury's appearance. It has been found that 49% of follow-up scans after operative management of severe liver injuries demonstrate new liver related complications, most of which require intervention [26]. Our patient underwent follow-up scans the fourth, tenth, twenty fourth and thirty first postoperative day.

Late complications of liver injury can occur following non-operative and operative management and include secondary bleeding, usually from pseudoaneurysms, infected collections and bile leaks. The majority of these complications can be dealt with by radiological intervention, with percutaneous drainage of collections, localization and temporary stenting of bile leaks by ERCP and angiographic embolization of secondary haemorrhages [27]. The percutaneous drainage of biloma with CT-guidance resulted in our patient's non operative treatment. ERCP (with sphincterotomy or bile duct stenting) was not performed in this patient due to the relevant morbidity of this procedure (acute pancreatitis, gastointestinal bleeding). A non-invasive alternative, MRCP, was performed instead; it did not indicate bile leak or other pathology, and further treatment was regarded unnecessary.

The most prevalent cause of morbidity and mortality, after the acute phase of penetrating injury, is infection. Penetrating abdominal trauma carries a high risk of serious infection because of immunosuppression from hemorrhage, transfusion and the high likelihood of intestinal injury. Presumptive antibiotic therapy should be initiated as soon as possible and should be continued for 24 hours. Prolonged courses of antibiotics offer no advantages over a 24-hour regimen [28]. As far as our patient is concerned, she received intravenous antibiotic therapy for a prolonged period due to the spinal canal inflammation.

In penetrating spinal trauma, X-rays, CT-scan [29] and MRI-scan [30] (when available and there is no metallic material) should be performed whereas Magnetic Resonance Angiography (MRA) is essential in specific injuries [31]. Our patient underwent X-rays of the vertebral column and MRI of the lumbar region after her initial resuscitation. The imagery did not indicate any cord change and our patient was considered to have good prognosis. However, the organic foreign debris in the spinal canal caused inflammation of lumbar vertebra and arachnoid membrane. This was confirmed on MRI of lumbar region on tenth postoperative day. Neurosurgeons removed it through laminectomy.

Current treatment of penetrating injury to the spine has been studied for gunshot wounds and for other penetrating trauma, such as stab wounds, with little difference in treatment goals. With any penetrating injury, treatment considerations include the neurological status of the patient, the stability of the spine related to the degree of bony and ligamentous injury, the path taken by the projectile or blunt object, the amount of canal compromise, and the location of retained fragments [32]. The nature and structure of the object, is also of great importance. A living organic body part, as the epithelium of swordfish bill, is loaded with bacteria and therefore is putrescible under all circumstances. Its removal is crucial and is strongly recommended, as learnt from that case. Prophylactic intravenous broad-spectrum antibiotic therapy has been used in the majority of patients' treatment of this type of trauma [1,3] - even with negative bacteriological results, as in our patient. According to literature, intravenous wide spectrum antibiotic therapy must be implemented to such patients for up to six weeks, to achieve an acceptable rate of cure. As a cost reduction exercise for patient, parenteral antibiotic administration on an outpatient basis or the use of oral antibiotics can be considered [33]. Our patient received intravenous antibiotic therapy for five weeks and left the hospital with the advice to continue her medication per os for three more weeks. Noticeably, use of steroid in penetrating injury to the spine has not demonstrated any benefit [34]. Our patient was not given any steroids.

Surgery in penetrating injury to the vertebral column is indicated for progressive neurological deficits, persistent cerebrospinal fluid leak or in the absence of neurological symptoms with radiological evidence of compression [35]. Although initial retrospective reports showed no benefits of surgical treatment compared with conservative management of penetrating spinal injuries [36], later data show that early extraction may reduce the incidence of infection, myelopathy and delayed neurologic loss [37]. However, the timing of surgical intervention in spinal injuries remains a subject of controversy. In the literature, both early and delayed surgical treatment of penetrating injuries to the spine with a remnant foreign body resulted to patients' neurological improvement [7,37,38]. Yet, we do not know to what extent the timing of surgical treatment was related to the neurological improvement. Moreover, we have to point out that management of a retained foreign body in a spinal penetrating injury even if desirable is often challenging, because removal of the object may cause neurological sequela. The reason mentioned above combined with the lack of experience in such patients treatment in our hospital was the main cause that made orthopaedics and neurosurgeons hesitant as far as foreign body's removal, from the posterior approach. The patient was a young woman lacking neurological deficits, after her spinal penetrating injury, therefore conservative management regarded appropriate. The subsequent inflammation of the spinal canal though, left the specialist with no other choice but the invasive one. The operation (laminectomy) was successful and the patient's clinical condition and radiological imaging improved rapidly.

## Conclusion

Although much has been written about the management of penetrating abdominal and spinal canal trauma, controversy remains over the optimal management.

The treatment of patients after penetrating abdominal trauma depends on institution- specific factors and on the type and location of the injury. Moreover, whenever surgery is indicated after penetrating spinal canal trauma, the objective of the treatment, the severity of neurological symptoms and the presence of systemic injuries all need to be considered.

## Consent

Written informed consent was obtained from the patient for the publication of the case report.

## Competing interests

The authors declare that they have no competing interests.

## Authors' contributions

DG, GNZ and GP designed research; DG, DV, SA, AK, PS and ENT performed research; CD and LP provided CT, MRI images and CT reconstruction images; DG wrote the paper

All authors read and approved the final manuscript.

## Pre-publication history

The pre-publication history for this paper can be accessed here:

http://www.biomedcentral.com/1471-2482/10/30/prepub
